# Culture and Drug Profiling of Patient Derived Malignant Pleural Effusions for Personalized Cancer Medicine

**DOI:** 10.1371/journal.pone.0160807

**Published:** 2016-08-22

**Authors:** Christian Ruiz, Stefan Kustermann, Elina Pietilae, Tatjana Vlajnic, Betty Baschiera, Leila Arabi, Thomas Lorber, Martin Oeggerli, Spasenija Savic, Ellen Obermann, Thomas Singer, Sacha I. Rothschild, Alfred Zippelius, Adrian B. Roth, Lukas Bubendorf

**Affiliations:** 1 Institute for Pathology, University Hospital Basel, Basel, Switzerland; 2 Pharmaceutical Sciences, Roche Innovation Centre Basel, Basel, Switzerland; 3 Department of Medical Oncology, University Hospital Basel, Basel, Switzerland; Universita degli Studi di Napoli Federico II, ITALY

## Abstract

**Introduction:**

The use of patients’ own cancer cells for in vitro selection of the most promising treatment is an attractive concept in personalized medicine. Human carcinoma cells from malignant pleural effusions (MPEs) are suited for this purpose since they have already adapted to the liquid environment in the patient and do not depend on a stromal cell compartment. Aim of this study was to develop a systematic approach for the in-vitro culture of MPEs to analyze the effect of chemotherapeutic as well as targeted drugs.

**Methods:**

MPEs from patients with solid tumors were selected for this study. After morphological and molecular characterization, they were cultured in medium supplemented with patient-derived sterile-filtered effusion supernatant. Growth characteristics were monitored in real-time using the xCELLigence system. MPEs were treated with a targeted therapeutic (erlotinib) according to the mutational status or chemotherapeutics based on the recommendation of the oncologists.

**Results:**

We have established a robust system for the ex-vivo culture of MPEs and the application of drug tests in-vitro. The use of an antibody based magnetic cell separation system for epithelial cells before culture allowed treatment of effusions with only moderate tumor cell proportion. Experiments using drugs and drug-combinations revealed dose-dependent and specific growth inhibitory effects of targeted drugs.

**Conclusions:**

We developed a new approach for the ex-vivo culture of MPEs and the application of drug tests in-vitro using real-time measuring of cell growth, which precisely reproduced the effect of clinically established treatments by standard chemotherapy and targeted drugs. This sets the stage for future studies testing agents against specific targets from genomic profiling of metastatic tumor cells and multiple drug-combinations in a personalized manner.

## Introduction

The main goal of personalized cancer medicine is to provide *“the right treatment for the right person at the right time”*, which increasingly relies on results from molecular analyses, such as genomic profiling [1, 2]. In the past, there have been multiple studies testing in-vitro assays with patients’ cancer cells to select the optimal drug treatment for patients with various tumor types including lung cancer and ovarian cancer [3–5]. Despite some promising results, these chemotherapy sensitivity and resistance assays (CSRAs) have not proved to be sufficiently reliable for clinical routine. Most of these assays were based on culture of tissue specimens from solid tumors which poses several technical challenges such as contamination with normal non-tumor cells or difficulties in disaggregation prior to culture which amongst other factors may have led to the observed lack of predictivity.

Today, the focus in personalized cancer medicine has moved towards the detection of novel predictive targets and the investigation of specific inhibitors of these targets [2, 6]. Targeted therapies based on a better molecular understanding of the disease have clearly improved the armamentarium of oncological therapies in various solid tumors. For example, in non-small cell lung cancer (NSCLC), administration of EGFR tyrosine kinase inhibitors (EGFR TKI) have shown high efficiency in the EGFR mutated subpopulation and have led to improved progression-free and overall survival (reviewed in [7]). Similarly, most patients whose tumors harbor an *ALK* gene rearrangement significantly respond to the ALK TKI inhibitors [8–10]. However, resistance to these targeted treatments is inevitable in most patients due to different mechanisms such as additional resistance mutations and/or activation of alternate signaling pathways, as it is well known for TKIs against EGFR and ALK [11–13]. The number of potential predictive markers will continue to increase due to powerful genome screening methods, such as next-generation sequencing, that are being applied in a number of ongoing large-scale cancer sequencing studies. However, translating potential driver mutations to an efficient drug against the related protein still remains a major challenge requiring preclinical testing of potential inhibitors in cancer samples in-vitro and in mouse models.

Whereas genomic profiles of carcinomas have mainly been acquired using solid tumor specimens, malignant pleural effusions (MPEs) have rarely been considered for these analyses. MPEs in the serosal cavities frequently occur in patients with metastatic lung, breast and ovarian cancer. The appearance of MPEs reflects the advanced or metastatic disease state beyond the organ of origin leading to significant therapeutic implications and worse prognosis [14]. The MPE samples are routinely processed in cytological laboratories and are subjected to the same diagnostic tests as for solid tumor specimens, such as morphological evaluation, immunocytochemical analysis (ICC) of protein markers, fluorescence-in situ hybridization (FISH) for detecting genomic rearrangements or amplifications, and sequencing specific mutations [15–17]. In contrast to most solid tumor biopsies MPEs have already adapted to the liquid environment and do not depend on stromal cells or vascularization. Therefore they may represent a promising cellular model representing the patient’s tumor. Of note, MPEs have been the source for many commercially available tumor cell lines. Previously, Basak et al used MPEs from lung cancer patients for the validation of putative cancer stem cells [18]. More recently, ascites cells from patients with high-grade serous carcinoma were used for exome sequencing in order to determine the clonal composition of their origin [19].

In this study, we aimed at establishing a robust system for the efficient testing of targeted therapeutics in MPEs from patients with solid tumors. For this purpose we used the xCELLigence culture system, which enables impedance based live-cell monitoring of proliferation, cytostasis or cytotoxicity [20]. Here, we show for the first time the utility of this approach in a proof-of-concept study. In the future, this functional system may support oncologists in tailoring therapy to individual patients based on the molecular profile of cancer cells in their MPEs.

## Materials and Methods

### Collection and processing of cells

All effusions processed in this study were acquired at the University Hospital Basel for routine diagnostics and were subjected to diagnostic analyses in the department of cytopathology of the Institute for Pathology. Effusions for culturing were processed as described [18]. Briefly, cell suspension was applied onto the Ficoll density gradient medium (Histopaque-1077, Sigma-Aldrich) and processed as recommended by the manufacturer. The cell layer (mononuclear cells) and the upper layer were subjected to a second centrifugation step and the resulting cell pellet was resuspended in Opti-MEM medium (Thermo-Fisher Scientific). For EpCAM based enrichment, the MACS (Miltenyi Biotec) system was used as recommended by the manufacturer. Briefly, 50ul of FcR Blocking Reagent and 50ul of CD326 (EpCAM) MicroBeads (Miltenyi Biotec) were added per 5x10^7^ cells and incubated for 30 minutes at 4°C. After washing, resuspended cells were passed through the MACS separator (Miltenyi Biotec). Release was performed by removing the column from the separator and flushing 5ml of buffer.

Resuspended effusion cells (from both Ficoll as well as MACS approach) were cultured in BD Primaria (BD Biosciences) flasks at 37°C and 5% CO2 with FBS (PAN-Biotech) or sterile filtered primary culture medium [18] and with the supplement of 1% Penicillin Streptomycin (Amimed, BioConcept, Allschwil Switzerland). Cell growth was monitored by usage of light microscopy and medium was replaced after every 5–7 days or when cells were splitted. For drug experiments, 1000x concentrated stock solutions of test compounds were prepared by dissolving powder in 100%DMSO (Sigma-Aldrich, St. Louis, MO) under sterile conditions. For cell treatment, stocks were diluted with culture medium and added to the cell culture wells at a final concentration as indicated at the graphs in the figures. Final solvent (DMSO) concentration was adjusted to 0.1% DMSO in the culture medium. Concentrations noted at the graphs are reflecting final compound concentration in solution of the cell culture medium. Compounds were obtained from Roche’s local sample repository. H522 and H3122 cell lines were maintained in RPMI-1640 supplemented with 10% heat inactivated fetal bovine serum (FBS, PAN-Biotech GmbH, Germany). All cells were cultured at 5% CO2 and 37°C.

This study was approved by the local ethical committee in Basel, Ethikkommission Nordwest- und Zentralschweiz (EKNZ), with the approval number EKBB 284/11.

### Cytospins and immunocytochemistry (TTF-1, Ber-EP4, Calretinin)

Before culture and during each splitting, cytospins were performed on a Cytospin 4 Cytocentrifuge (Thermo Scientific Limited) at 800rpm for 2 minutes. Cytospins were fixed in Delaunay solution and standard immunocytochemistry procedures were applied using the automated BOND-MAX system (Leica Biosystems, Muttenz, Switzerland). Antibodies used: TTF-1 (Clone 8G7G3/1, 1:100 dilution, pretreatment: ER1, 5 min 80°C, Neomarkers/Thermo Scientific), Ber-EP4 (clone M804, 1:400 dilution, DAKO) and Calretinin (Clone Cal6, 1:100 dilution, pretreatment: ER1 10 min, 80°C, Leica). All cytospins were evaluated by board certified pathologists (LB, TV).

### Monitoring of cell growth using real-time cell analysis

Cell growth experiments were performed by usage of the real-time cell analysis system xCELLigence (ACEA) as previously described [20]. Briefly, 10’000 effusion cells were plated per well (96-well format) and their growth was continuously monitored for at least 90 h in 15 min intervals. All xCELLigence experiments were performed in triplicates. One day after seeding, test compounds were added to the culture in the specified concentrations.

## Results

### Usage of a real-time monitoring system for drug target experiments with commercially available carcinoma cell lines

**Fig 1:**
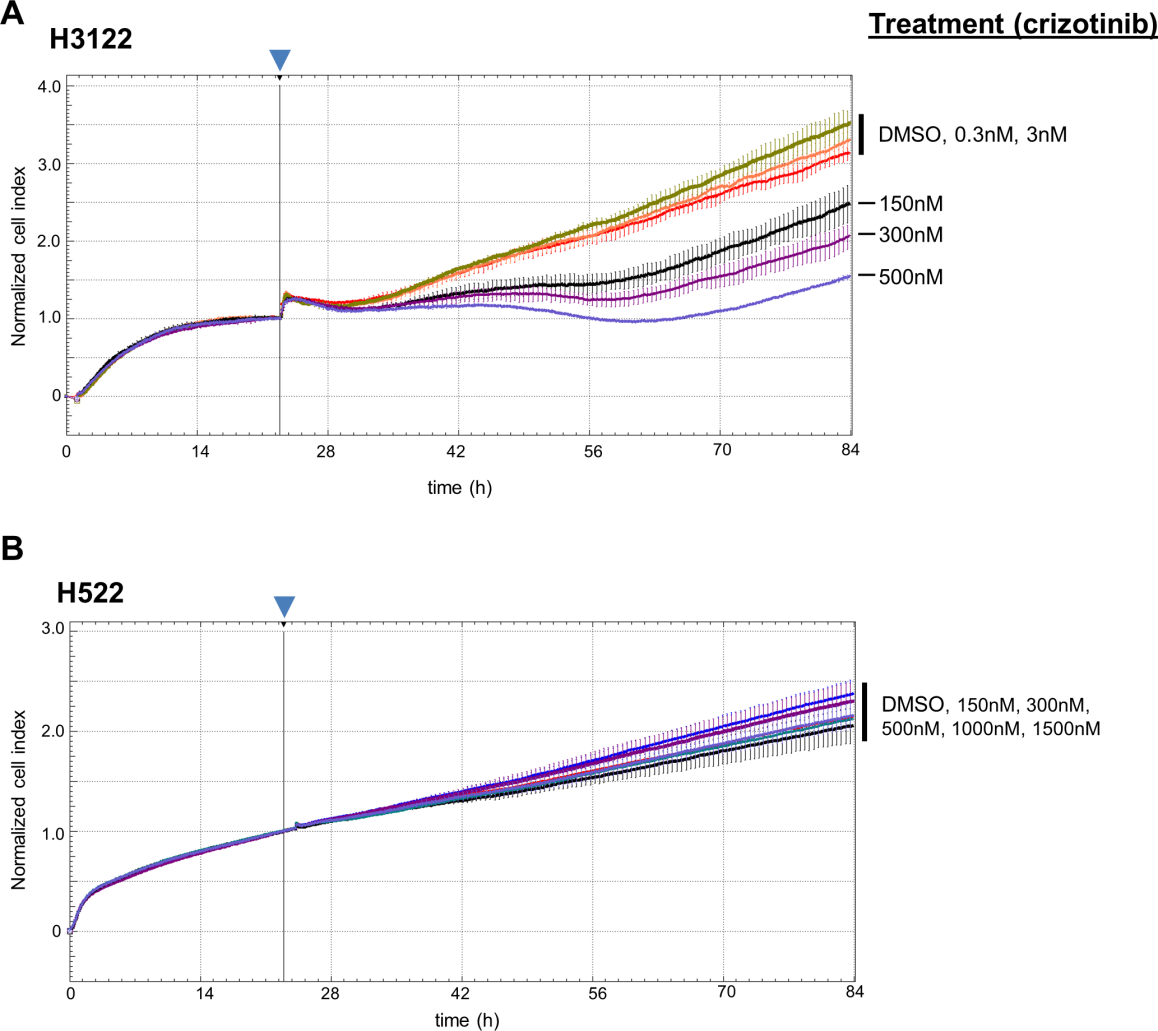
Measurement of the growth inhibitory effect of the TKI crizotinib in real-time. A. H522 cells, which are wild-type for ALK do not respond to crizotinib treatment. B. The ALK rearranged H3122 lung cancer cells show high sensitivity for even low doses of crizotinib. Arrow head points towards time point used for normalization.

As shown previously, the xCELLigence system is well-suited to analyze drug effects on cell proliferation, cytostasis and cytotoxicity in real-time [20]. In contrast to single end-point assays, the availability of drug effect profiles over the whole experimental period allows building of dynamic growth curves. In order to assess the utility of this system for analysis of targeted therapies, we subjected two established non-small cell lung cancer cell lines H522 and H3122 to crizotinib treatment, a tyrosine kinase inhibitor (TKI) with activity against ALK, ROS1 and CMET [21–23]. Whereas H3122 cells harbor a rearrangement in the *ALK* gene, H522 cells are wild-type for *ALK*, *ROS1* and *CMET* [21, 24]. We treated the *ALK*-rearranged cell line H3122 and the control cell line H522 with different concentrations of crizotinib. As expected, treatment with crizotinib led to a significant growth reduction in H3122 cells (Fig 1A), but not in the control H522 cells (Fig 1B). Whereas lower concentrations of crizotinib (0.3nM/3nM) were not effective, concentration of 150nM or above significantly reduced growth of H3122 cells, but not of H522 cells. Thus, our data suggest that effects of targeted drugs such as crizotinib can be reliably assessed using this real-time monitoring system.

### Preparation and culturing of pleural effusions over time

**Fig 2:**
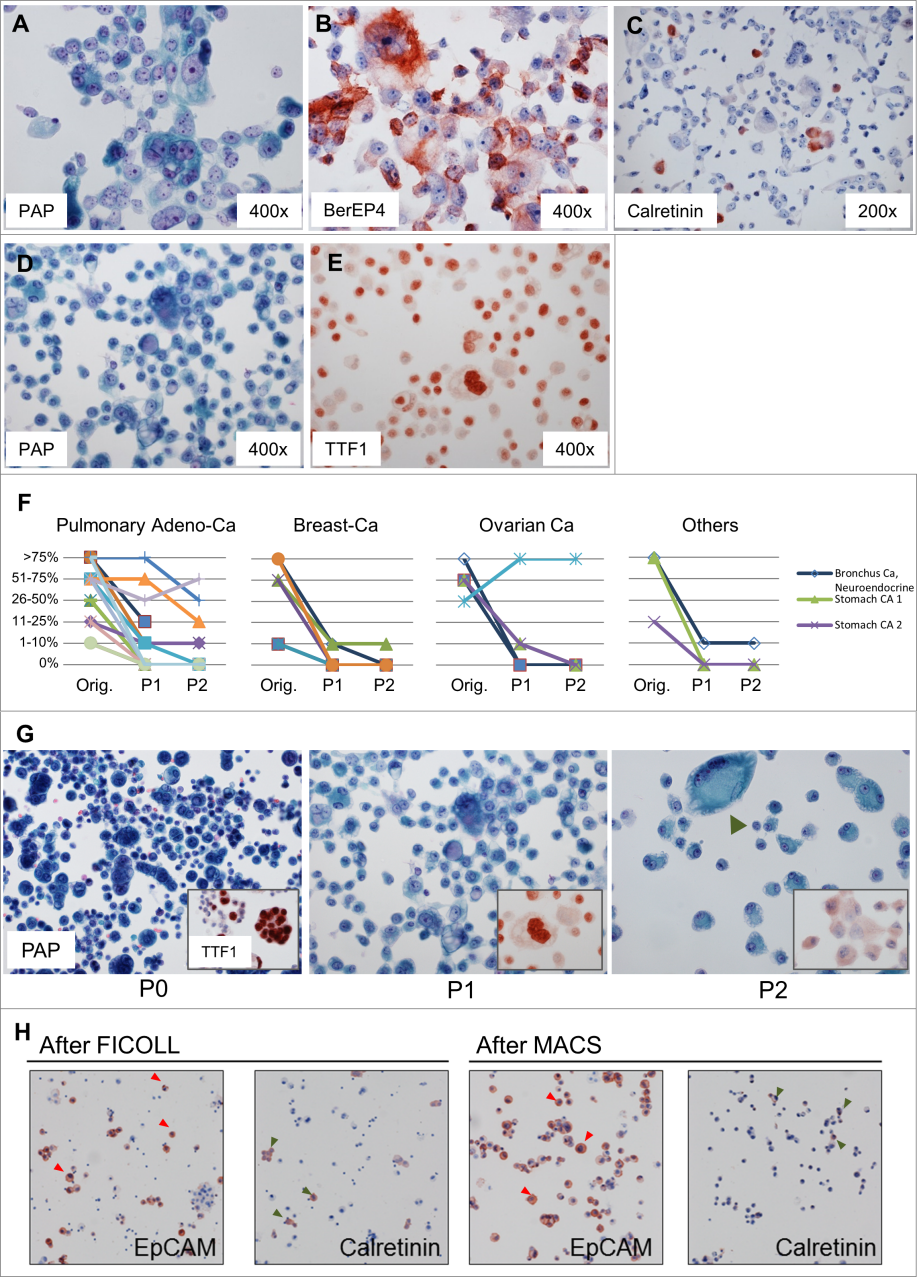
Determining tumor cell content in cultured malignant effusions. A-C. Second passage (P2) of a malignant effusion of a metastatic ovarian carcinoma. BerEP4 (B) and Calretinin (C) were used as epithelial and mesothelial markers, respectively. In this case, the number of mesothelial cells in passage 2 was very low (1–10%) (This case corresponds to the light blue line in the histogram of 2G, Ovarian Ca.). D-E. First passage (P1) of a malignant pleural effusion of an adenocarcinoma of the lung. TTF1 (nuclear) was used as specific marker for epithelial cells. F. In most of the effusions, relative tumor cell content decreased during the first passages in culture. Y-axis denotes relative tumor content as determined by the pathologist. G. Example of the tumor cell decrease in a malignant effusion from a patient with lung adenocarcinoma. In P0 and P1 most of the cells were from a pulmonary adenocarcinoma, as shown by TTF1 nuclear positivity (small box). In contrast, cells in P2 were mostly of non-epithelial origin (no specific nuclear TTF1 staining). Of note, mesothelial cells can change their morphology in culture and show pronounced atypia, and may be mistaken for tumor cells (green arrowhead). H. Substantial increase in tumor cells (EpCAM positive) and decrease in mesothelial cells (Calretinin positive) after enrichment with EpCAM antibody coated magnetic beads (MACS). Red arrowheads point towards epithelial tumor cells, green arrowheads towards mesothelial (non-neoplastic) effusion cells.

To determine optimal conditions for culturing pleural effusions and real-time monitoring of their growth, we tested different growth medium supplements, coatings of cell culture dishes and tumor enrichment approaches. Patient-derived pleural effusions are adapted to the liquid environment in vivo, and in order to best mimic their original environment, the use of commercial fetal bovine serum might be considered to be sub-optimal. We thus followed the procedure recommended by Basak et al and separated non-nucleated cells from pleural effusions via Ficoll-gradient and used sterile-filtered effusion supernatant instead of commercially available serum [18]. We confirmed that medium supplemented with supernatant from the same patient led to significantly increased growth and viability of pleural effusions compared to effusions grown in medium supplemented with 10% FCS (S1 Fig). Furthermore, we used commercially available surface-modified polysterene plates to increase initial adherence of patient-derived effusion cells (BD Primaria plates from BD Biosciences, S1 Fig). In order to monitor tumor cell content over several passages, we performed *Papanicolaou*-staining of cytospin preparations before culture (as part of diagnostic routine) and after each passage. We estimated tumor cell content by cytology and by immunocytochemistry (ICC) using markers previously confirmed to be positive when measured as part of routine clinical diagnostic procedure. In general, EpCAM (epithelial cell adhesion molecule, BerEP4) (Fig 2B) and Calretinin (Fig 2C) were used as pan-epithelial and mesothelial markers, respectively, and if necessary, additional tumor specific markers were analyzed, such as TTF1 (thyroid transcription factor 1) (Fig 2E) in effusions originating from lung cancer patients. We observed a dramatic decrease of relative tumor cell content after few passages in almost all of the malignant pleural effusions analyzed (Fig 2F). This included effusions from lung, breast, ovarian, stomach and colon cancer. This decrease was accompanied by a relative increase in mesothelial cells, which with increasing culture time outgrew epithelial cells (Fig 2G). We therefore used magnetic beads coated with EpCAM antibodies as an optional additional step to enrich for cells of epithelial origin. As depicted in Fig 2H, enrichment with magnetic beads led to a reduced number of mesothelial cells in the culture in comparison to cultures treated with Ficoll-gradient alone. Thus, this protocol enabled the use of pleural effusions in vitro, even when derived from patient samples with low tumor cell content. Of note, a very high tumor cell proportion was considered ideal when using the system for tests with targeted therapeutics to minimize the impact of non-responding normal mesothelial cells in the culture.

### Treatment of malignant cells in pleural effusions with cytotoxic agents and targeted therapeutics

**Fig 3:**
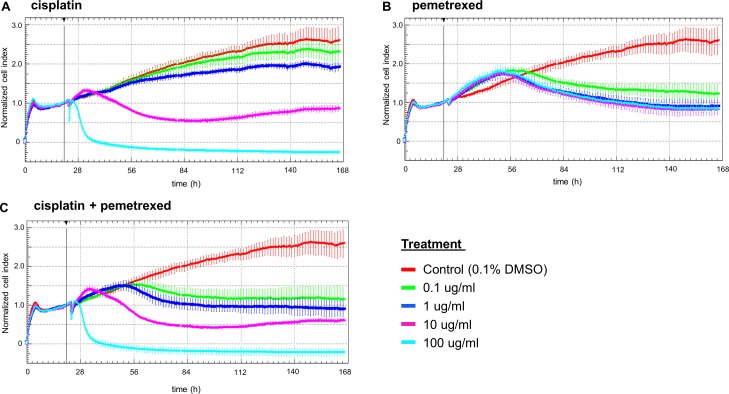
Treatment of effusions with cytotoxic agents. Treatment of a malignant pleural effusion from pulmonary adenocarcinoma with different concentrations of cisplatin (A), pemetrexed (B) and a combination thereof (C).

Similar to the cell line experiments described above (Fig 1), we treated patient-derived MPEs with distinct cancer origins with cytotoxic agents based on the standard of care in the clinic setting and specific recommendation from medical oncologists. As expected, treatment of pleural effusions with the cytotoxic agents cisplatin or pemetrexed or a combination thereof led to a dose-dependent reduction of cell growth in malignant pleural effusions from pulmonary adenocarcinomas (Fig 3A–3C). The same effect was observed for MPEs from other tumor types (data not shown), as well as an effusion from a malignant mesothelioma (S2 Fig) and in a benign effusion (normal control, non-malignant mesothelial cells) (S3 Fig). The real-time monitoring system allowed us to determine the latency time of the drug effect on cell growth. The growth-reducing effect of cisplatin started before 40h (Fig 3A: 10ug/ml), whereas for pemetrexed first effects on cell growth were detected after 50h only (Fig 3B: 10ug/ml). This difference was expected since cisplatin directly targets the DNA and thereby induces apoptosis, whereas pemetrexed acts as an inhibitor of enzymes involved in the purine and pyrimidine synthesis process.

**Fig 4:**
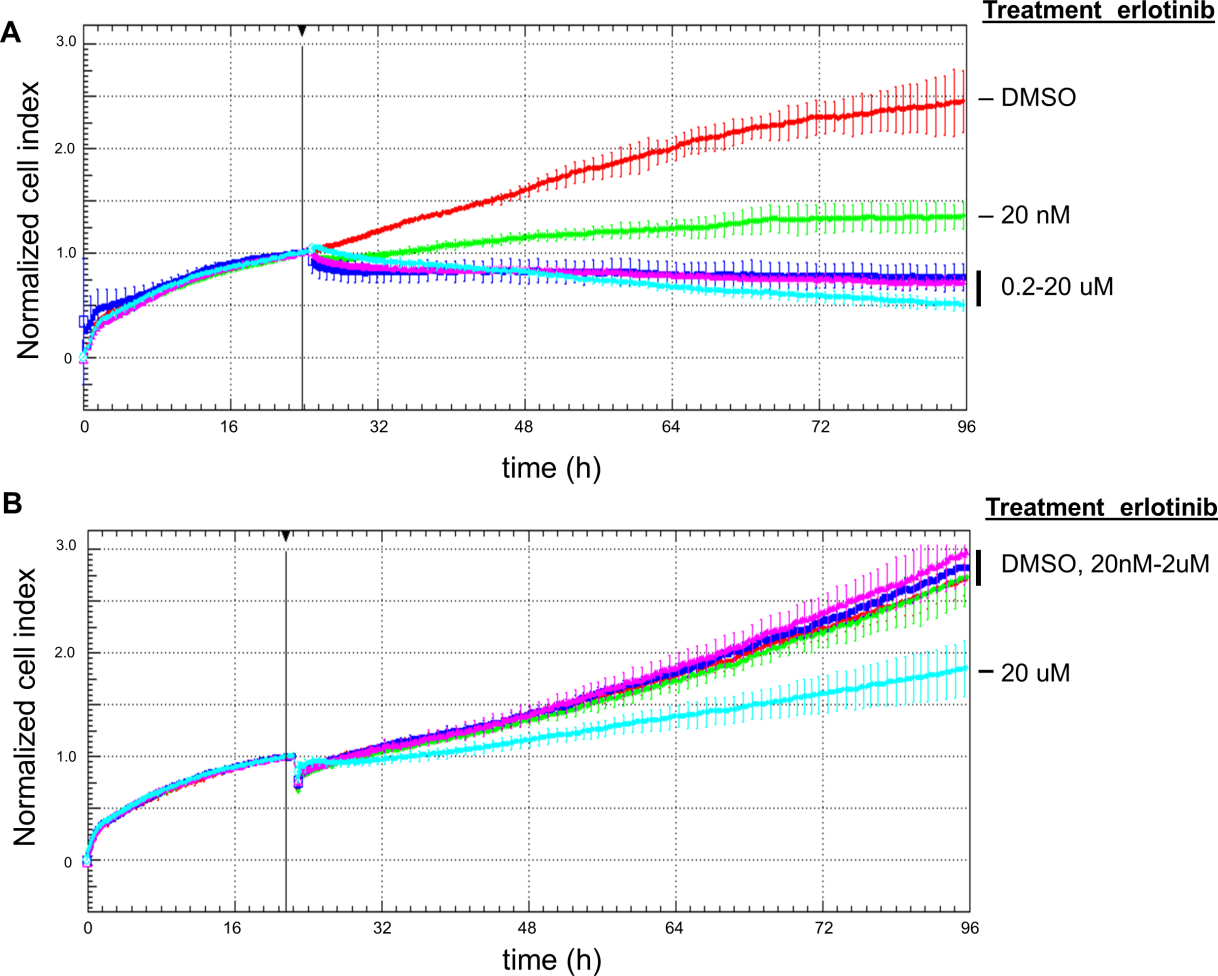
Treatment of lung cancer MEs with the TKI erlotinib based on their genomic profile. Only the effusion with the activating *EGFR* gene mutation (A) responded to treatment with the tyrosine kinase inhibitor erlotinib, but not the effusion with wild-type *EGFR* (B).

We next interrogated the usability of our system for treatment of patient-derived malignant effusions with targeted therapeutics. For this purpose, we selected MPEs with genomic markers known to qualify for treatment with a specific targeted drug. The first malignant pleural effusion was from a 73 year old male patient with metastatic adenocarcinoma of the lung, positive for TTF1 and BerEP4. Routine diagnostic sequencing procedure revealed an activating mutation in the *EGFR* gene (p.E476-T751 delinsA, exon 19) but no mutations in *KRAS*, *BRAF* and *HER2*, as well as no *ALK* gene rearrangement as determined by FISH. Based on these molecular characteristics, we assumed this lung cancer to be responsive to an EGFR-TKI. In addition, and as a control, we selected a pleural effusion from a 62 year old patient with metastatic TTF1 positive adenocarcinoma of the lung. Genomic analysis revealed neither an *ALK* gene rearrangement nor *EGFR*, *BRAF* or *HER2* gene mutations, but an activating point mutation of the *KRAS* gene (p.G12C, exon 2) and therefore this tumor could be considered a non-responder case to EGFR-TKI therapy. We thus processed both pleural effusions as described above and treated the cultured cells with the EGFR TKI erlotinib. The pleural effusion with the *EGFR* mutation showed a dose-dependent growth reduction upon treatment with erlotinib (Fig 4A). Importantly, this was not the case for the MPE of the *KRAS* mutated but *EGFR* wild-type adenocarcinoma (Fig 4B). Concordantly, reviewing of the clinical follow-up of the patient with the *EGFR* mutant effusion (Fig 4A) showed a partial response (>50%) by computer tomography (CT) scan after the first two months of treatment with erlotinib. These findings provide further evidence that our system of in-vitro culturing of patient-derived malignant effusions can assess the response to specific inhibitors (targeted therapeutics) based on their genomic vulnerabilities.

## Discussion

The use of genomic markers for therapy decisions has become a fundamental part of personalized medicine in clinical oncology. Based on the presence of druggable somatic driver mutations, patients are selected for specific therapeutic treatment. The number of such targets and corresponding drugs is continuously increasing. However, a full translation of somatic driver mutations into an effective, tailor-made therapy for an individual patient is still a challenge.

We therefore aimed to establish a robust real-time in vitro system that can be used for the testing of targeted inhibitors on patients’ cancer cells obtained from malignant effusions, which are a common manifestation of metastatic disease. In line with previous publications monitoring drug effects on cell growth and survival, we showed that the impedance based real-time monitoring system can reproduce known effects of chemotherapeutic agents and targeted inhibitors on commercially available cell lines [20]. Here, we could successfully develop a protocol for culturing and drug testing of primary cultures from malignant pleural effusions of pulmonary adenocarcinomas and a malignant mesothelioma as demonstrated by experiments with standard chemotherapy and targeted inhibitors selected based on the genomic profile of tumor cells. Only the lung cancer effusion with the sensitive *EGFR* mutation, but not the *EGFR* wild-type one, responded to erlotinib in a dose-dependent manner, which confirms the relevance of this new in vitro test system. The targeted nature of the treatment was emphasized by the lack of an effect in control experiments using *EGFR* wild-type cell lines or primary cultures. In contrast, the effect of standard chemotherapy did not differentiate between normal proliferating mesothelial cells from benign effusions and carcinoma cells, both of which are vulnerable to unspecific inhibition of proliferation and DNA synthesis. This study may be considered as proof-of-concept since we could reproduce the treatment effect known from clinical practice.

Malignant effusions have rarely been considered as a substrate for basic research although they represent a suitable model for studying the characteristics and behavior of metastatic carcinoma. Based on the previous work by Basak et al [18], we have optimized the (pre)culturing conditions and adapted them for their use in drug testing experiments with the real-time impedance-based growth monitoring system xCELLigence. Whereas culture and drug treatment of commercially available cell lines was straight forward, culturing of primary effusion cells required substantial modifications. Similar to Basak et al, we experienced higher growth rates when sterile-filtered MPE supernatant was used as growth serum [18]. The use of surface-modified polystyrene cell culture dishes further increased the number of attached effusion cells. One of the major issues of solid tissue culturing, besides tissue disaggregation, is the prominent outgrowth of normal fibroblasts when cells from solid tissue specimens are placed into culture. As expected, we did not detect any fibroblasts, but after few passages mesothelial cells had outgrown the carcinoma cells to such an extent that a drug testing would not have been representative of the tumor. Importantly, morphological analysis is often not reliable enough to discern carcinoma and mesothelial cells, since the morphology of both carcinoma cells and mesothelial cells often changes in culture [25]. The resulting morphological overlap between benign and malignant cells in culture might be partly due to the high proliferative activity of the mesothelial cells and epithelial-mesenchymal transition (EMT) of the carcinoma cells. Thus, we used immunocytochemistry with cancer-specific markers in effusions such as TTF-1 or the epithelial marker EpCAM combined with the mesothelial marker calretinin to better quantify tumor cells across passages [25, 26]. Enrichment of tumor cells can be achieved by a positive selection strategy using EpCAM antibody-coated magnetic beads before placing the effusion cells in culture. This optional step reduces the number of mesothelial cells to negligible levels even after passaging. However, other methods, such as fluorescence activated cell sorting (FACS) or a negative selection strategy by actively removing mesothelial cells with antibodies may also be considered. Importantly, the aim of this study was not the optimization of parameters for long-term culture of primary effusion cells, but their growth for a limited time period (one to three weeks) in order to perform the analyses and the drug experiments. The time needed for cytological and predictive biomarker analysis ranges from a few days (immunocytochemistry or FISH) to up to two weeks (mutation analysis). Thus, freezing the tumor cells to initiate culture at the time of completed biomarker testing might also be considered. Otherwise, continuous growth could lead to a decreasing proportion of tumor cells while waiting for the results of mutation profiling. In fact, short-term (up to 3 months) DMSO-based freezing and re-culturing of primary effusion cells did not negatively affect vitality of the tumor cells in our study (data not shown). The long-term storage of patients’ malignant effusion might also be interesting since it would allow for biomarker and drug sensitivity testing in case novel targeted treatments should become available at a later point in time.

The concept of culturing tumor cells from patients in order to test their response to therapies has been intensively discussed and assayed over the last decades [27]. Commercially available immortalized cell lines are not suited for this purpose given the heterogeneity of cancer in general and the acquired genetic changes after months or years of growing in culture [28]. Further, the majority of the cell lines used in research are matched based to the tumor subtype and not to the molecular profile as recently shown for ovarian cancer [29]. Culturing of solid tumor biopsies may be regarded as an optimal approach, but they have to be dissociated and often depend on their microenvironment with normal stromal cells or vascularization. Furthermore, the number of cells from a solid tumor specimen remaining after resection and histological examination is very low and would restrict any potential in vitro experiments to a minimum–if possible at all. These limitations make them less suitable for efficient drug testing analyses in daily practice. On the other hand, major advances have been made in developing more sophisticated cell culturing systems such as the hanging drop mechanism [30] or the bioreactor [31]. It remains to be seen how these systems can be used in a routine clinical setting. Another approach currently under consideration is xenografting patient tumor tissue for drug testing (reviewed in [32]). Whereas this approach is very interesting for research purposes, its routine clinical applicability may be challenging due to the time required until cells start to grow and thus testing can be initiated. More importantly, the very low tumor take rates for certain tumor types may make drug testing using this approach impossible [33]. We believe that our approach using patients’ MPEs could be superior compared to approaches based on use of solid tumor tissue, but further studies are needed in order to validate this in-vitro testing. Importantly, regardless of the in-vitro system chosen, monitoring and quantifying the proportion of tumor cells in culture is indispensable. For the here proposed system, ICC with cancer-specific markers (such as TTF-1 or BerEP4) as well as a mesothelial marker (such as calretinin) on cytospins at the tie of initial culture and after each splitting is recommended.

Taken together, in this proof-of-concept study, we have developed a new system for in vitro sensitivity testing of primary cultures from malignant effusions that overcomes some of the key limitations of previous approaches due to technical improvements allowing precise measurement of cell growth in a real-time manner. While only a limited number of effusions with specific targeted therapeutics have been tested so far, the data presented here are very encouraging and underline the potential of this approach. Further studies using larger numbers of malignant effusions with druggable mutations and a correlation with the clinical course of the affected patients are therefore needed. It is anticipated that in the future this system could be used in cooperation with clinical oncologists as part of a personalized medicine approach to support selection of targeted therapies specific to an individual. Of note, the here proposed approach can only be applied to patients with a MPE. Additionally, the system provides an easy tool with a reasonable throughput to test a multitude of novel drug combinations in a personalized medicine manner where a patient with a MPE could directly benefit from. This might not be a priority for some currently approved treatments with EGFR or ALK inhibitors, given the high response rates. However, with the upcoming large-panel NGS mutation testing in routine practice, oncologists are confronted with an increasing number of mutations whose predictive effect in individual patients is not optimal characterized. In-vitro testing of new inhibitors based on the profile of the patient’s tumor could be used for decision making for early access programs or clinical trials. In addition, not yet approved targeted drugs and combinations thereof could be safely tested using these malignant effusions showing respective mutations to estimate the potential clinical benefit of such drugs if provided within compassionate use programs.

## Supporting Information

S1 Fig Optimization of growth conditions. A. Increased growth of pleural effusion cells when patient-derived effusion supernatant was added to the medium. B. Increased adherence of pleural effusion cells when surface modified polystyrene culture plates was used.
(TIF)

S2 Fig Malignant mesothelioma. Treatment of a malignant mesothelioma with different concentrations of cisplatin (A), pemetrexed (B) and a combination thereof (C).
(TIF)

S3 Fig Normal mesothelial cells. Treatment of normal mesothelial cells with pemetrexed (A) and cisplatin (B).
(TIF)
