# Supplementary figures and images for: Culture and Drug Profiling of Patient Derived Malignant Pleural Effusions for Personalized Cancer Medicine
Source: PLoS One. 2016 Aug 22;11(8):e0160807. doi: 10.1371/journal.pone.0160807 (PMC4993361; doi:10.1371/journal.pone.0160807)

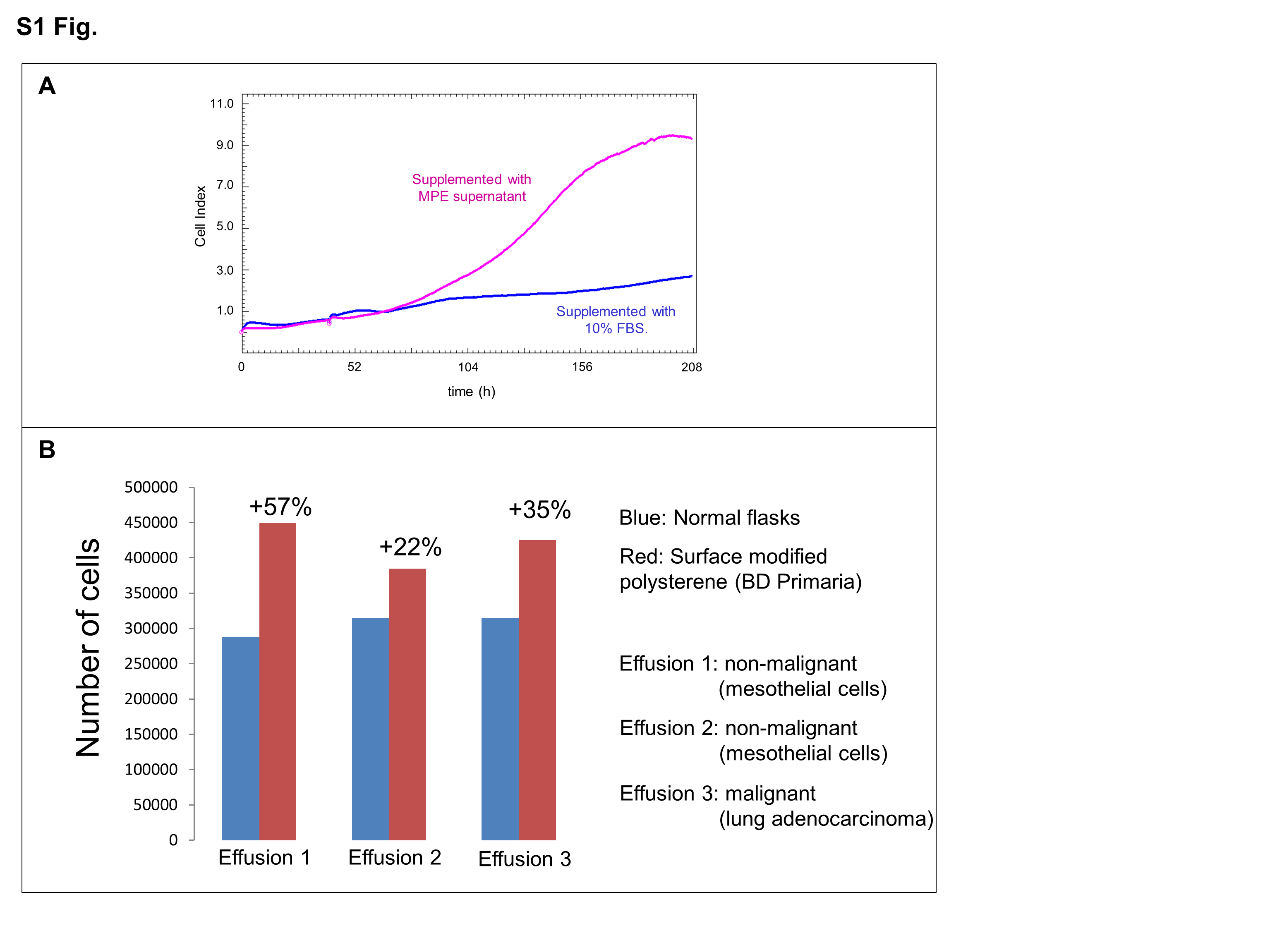

Supplement: S1 Fig — A. Increased growth of pleural effusion cells when patient-derived effusion supernatant was added to the medium. B. Increased adherence of pleural effusion cells when surface modified polystyrene culture plates was used. (TIF) [file pone.0160807.s001.tif]

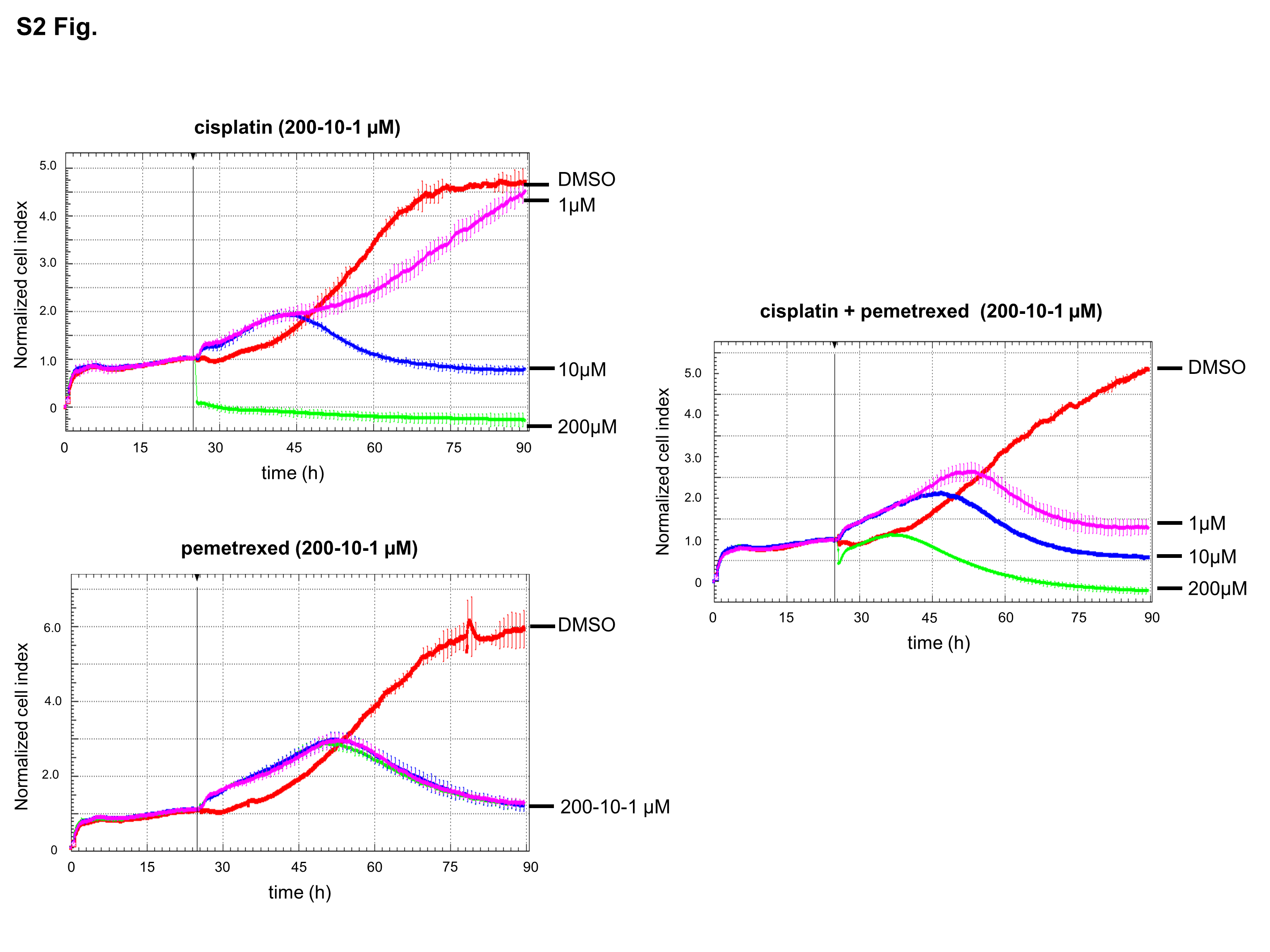

Supplement: S2 Fig — Treatment of a malignant mesothelioma with different concentrations of cisplatin (A), pemetrexed (B) and a combination thereof (C). (TIF) [file pone.0160807.s002.tif]

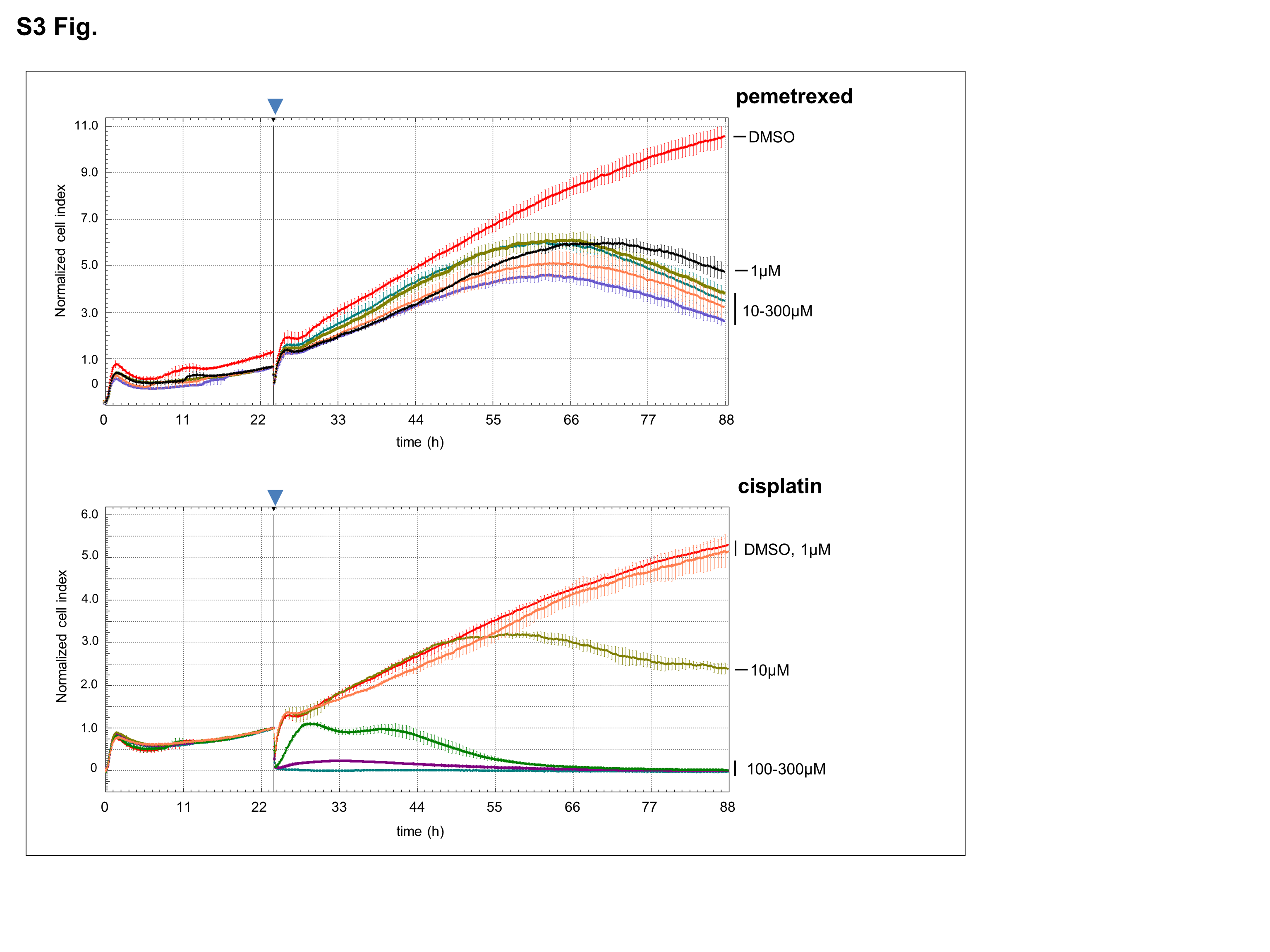

Supplement: S3 Fig — Treatment of normal mesothelial cells with pemetrexed (A) and cisplatin (B). (TIF) [file pone.0160807.s003.tif]
